# Circulating gamma-glutamyl transferase and development of specific breast cancer subtypes: findings from the Apolipoprotein Mortality Risk (AMORIS) cohort

**DOI:** 10.1186/s13058-017-0816-7

**Published:** 2017-03-06

**Authors:** Lydia Shackshaft, Mieke Van Hemelrijck, Hans Garmo, Håkan Malmström, Mats Lambe, Niklas Hammar, Göran Walldius, Ingmar Jungner, Wahyu Wulaningsih

**Affiliations:** 1 Division of Cancer Studies, Cancer Epidemiology Group, King’s College London, Faculty of Life Sciences & Medicine, Guy’s Hospital, 3rd Floor, Bermondsey Wing, London, SE1 9RT UK; 2 Regional Cancer Centre, Uppsala, Sweden; 3 Unit of Epidemiology, Institute of Environmental Medicine, Karolinska Institutet, Stockholm, Sweden; 4 Department of Medical Epidemiology and Biostatistics, Karolinska Institutet, Stockholm, Sweden; 5 AstraZeneca R&D, Mölndal, Sweden; 6 Unit of Cardiovascular Epidemiology, Institute of Environmental Medicine, Karolinska Institutet, Stockholm, Sweden; 7 Department of Medicine, Clinical Epidemiological Unit, Karolinska Institutet and CALAB Research, Stockholm, Sweden; 8 MRC Unit for Lifelong Health and Ageing at University College London, London, UK

**Keywords:** GGT, Breast cancer, Glucose, Triglycerides, Prospective study

## Abstract

**Background:**

Different etiological pathways may precede development of specific breast cancer subtypes and impact prevention or treatment strategies. We investigated the association between gamma-glutamyl transferase (GGT) and development of specific breast cancer subtypes based on oestrogen receptor (ER), progesterone receptor (PR) and HER2 status.

**Methods:**

We included 231,283 cancer-free women in a Swedish cohort. Associations between GGT and breast cancer subtypes were investigated with nested case–control and case–case analyses. We used logistic regression models to assess serum GGT in relation to breast cancer subtype, based on individual and combined receptor status.

**Results:**

Positive associations were found between serum GGT and development of ER+, ER− and PR+ breast cancers compared to controls (odds ratio (OR) 1.14 (95% confidence interval (CI) 1.08–1.19), 1.11 (1.01–1.23) and 1.18 (1.12–1.24), respectively) and of ER+/PR+ tumours. We found inverse associations between GGT levels and PR− breast cancers compared to PR+ (OR 0.87 (0.80–0.95)), between ER+/PR− tumours compared to ER+/PR+ tumours and between ER−/PR−/HER+ compared to ER+/HER2 or PR+/HER2 tumours (OR 0.55 (95% CI 0.34–0.90).

**Conclusion:**

The observed associations between pre-diagnostic serum GGT and different breast cancer subtypes may indicate distinct underlying pathways and require further investigations to tease out their clinical implications.

**Electronic supplementary material:**

The online version of this article (doi:10.1186/s13058-017-0816-7) contains supplementary material, which is available to authorized users.

## Background

Increased levels of serum gamma-glutamyl transferase (GGT) is a marker of oxidative stress [1], which may lead to tumour development, progression and metastasis [2] through modification of signalling pathways and DNA damage [2–4]. We previously showed an association between elevated serum GGT and risk of breast cancer in Swedish women [5], which were supported in a large systematic review and meta-analysis [6]. However, the association between circulating GGT and breast cancer subtype is unclear. Development of specific breast cancer subtypes significantly impacts therapeutic decisions and prognosis, but their underlying mechanisms remain elusive. To assess the role of oxidative stress, we now investigated the association between pre-diagnostic GGT and breast cancer subtype in nested case–control and case–case studies in a large Swedish cohort.

## Methods

### Study population

The AMORIS study has been described in detail elsewhere [5, 7–9]. This cohort includes 812,073 individuals who underwent laboratory examination at the Central Automation Laboratory in Stockholm between 1985 and 1996 [9]. The study complied with the declaration of Helsinki and was approved by the Ethics Review Board of the Karolinska institute.

From the AMORIS cohort we identified 231,283 cancer-free women aged 20 years or older with baseline measurements of serum GGT. These women were followed until they developed breast cancer, died, emigrated, or until the end of the study (31 December 2011), whichever came first. A total of 10,861 breast cancers (4.7%) were diagnosed during follow-up. Among them, 6934 (63.8%) had available information on oestrogen receptor (ER) status, 7145 (65.8%) had information on progesterone receptor (PR) status, and 2197 (20.2%) had additional information on HER2 status. A nested case–control study was performed where for each case with information on receptor status, we used incidence density sampling to select ten controls among all women in the cohort who were alive and did not have breast cancer at the time of diagnosis of the case. Cases and controls were matched for age group (less or more than 50 years old) as an indicator for menopausal status [10] because menopausal status was only available for cases. The same sets of cases were included in the case–case analysis.

### Breast cancer diagnosis and subtype

We classified breast cancer subtype based on ER and PR and their combinations. In the subgroup with information on HER2, we defined four tumour subtypes (ER+/HER2− or PR+/HER2−, ER+/HER2+ or PR+/HER2+, ER−/PR−/HER2+, and ER−/PR−/HER2− (triple negative)) as previously described (Additional file 1: Figure S1) [11]. These subtypes share similar profiles with molecular phenotypes luminal A, luminal B, HER2 type and triple negative [12, 13].

### Assessment of exposures and covariates

All laboratory analyses were performed by automated techniques at the CALAB laboratory, Stockholm, Sweden. GGT (U/L) was determined using the reference method recommended by the International Federation of Clinical Chemistry and Laboratory Medicine (IFCC) [5, 14]. The coefficient of variation was ≤6.0%. Samples were prospectively measured prior to assignment to cases or controls. Levels of GGT were skewed and logarithmically transformed. We additionally categorised GGT into quartiles.

From the registry linkage in AMORIS [5, 9], we collected information on socioeconomic status, education level, parity, menopausal status at diagnosis, and comorbidities using Charlson co-morbidity index (CCI) [15, 16]. Serum triglycerides and glucose were measured enzymatically [17].

### Statistical analysis

In the nested case–control analysis, we used conditional logistic regression models to assess any association between log-transformed and quartiles of GGT and overall and specific breast cancer subtypes. A test for trend was performed by using GGT quartiles as an ordinal scale. We estimated odds ratios (ORs) of ER and PR status individually compared to matched controls based on these measures of serum GGT. Subsequently we compared GGT levels of cases with controls based on combined ER and PR subtypes.

We further conducted a case–case analysis to compare different breast cancer subtypes [18]. Binary and multinomial logistic regression models were used to assess log-transformed levels and quartiles of GGT in relation to breast cancer subtype, both by individual ER or PR status, combined ER/PR status and ER/PR/HER2 status. Since ER and PR status was available since follow-up started and the information of HER2 status was only available after 2006, we performed a sensitivity analysis only including cases with complete information on the three receptors.

All models were adjusted for age, socioeconomic status, education and parity, and time interval between GGT measurement and diagnosis. We additionally controlled for menopausal status in the case–case analysis. Adjustment for CCI was performed to take into account existing co-morbidities [1, 5, 19, 20]. We further adjusted for serum glucose and triglycerides to reduce potential confounding from metabolic disorders [11, 21–23]. All analyses were conducted with Statistical Analysis Systems (SAS) release 9.4 (SAS Institute, Cary, NC, USA).

## Results

### Case-control analysis

The mean age of diagnosis was 61.68 years and most women were postmenopausal (Additional file 1: Table S1). Serum GGT was slightly higher in cases than in controls. Overall, higher log-transformed GGT correlated with higher odds of any breast cancer (OR 1.13, 95% confidence interval (CI) 1.08–1.19). There was a positive association between continuous levels of GGT and development of ER+, ER−, and PR+ breast cancers (Table 1), with the strongest association seen for PR+ tumours (OR 1.18, 95% CI 1.12–1.24). Results were similar with GGT quartiles.

**Table 1:** Conditional logistic regression model with breast cancer subtype as main outcome

GGT U/L	Number of hormone receptor-positive cases	Number of controls	OR (95% CI)	Number of hormone receptor-negative cases	Number of controls	OR (95% CI)
ER status versus control						
GGT log	5939	59390	1.14 (1.08–1.19)	1295	12950	1.11(1.01–1.23)
0–11.40	1310	13100	1 (Ref)	296	2960	1 (Ref)
11.40–15.00	1424	14240	1.08(1.00–1.17)	308	3080	1.04(0.88–1.23)
15.00–21.60	1585	15850	1.11(1.03–1.20)	340	3400	1.08(0.92–1.28)
≥21.60	1620	16200	1.25(1.15–1.35)	351	3510	1.21(1.02–1.43)
P_trend_			<0.0001			0.03
PR status versus control						
GGT log	4938	49380	1.18(1.12–1.24)	2207	22070	1.06(0.98–1.15)
0–11.40	1068	10680	1 (Ref)	512	5120	1 (Ref)
11.40–15.00	1175	11750	1.09(1.00–1.19)	531	5310	1.04(0.91–1.18)
15.00-21.60	1324	13240	1.16(1.06–1.26)	580	5800	1.02(0.90–1.16)
≥21.60	1371	13710	1.33(1.22–1.45)	584	5840	1.11(0.97–1.26)
P_trend_			<0.0001			0.18

Hormone receptor-positive or -negative cases referred to cancer subtypes based on individual ER or PR status. Controls without breast cancer were the referent groups. All models were adjusted for age at diagnosis, socioeconomic status, education, parity, CCI, and interval between measurement and cancer diagnosis or control selection date

We subsequently investigated the association between serum GGT and combined receptor subtypes of breast cancer based on ER/PR status. A significant positive association between log-transformed GGT and development of ER+/PR+ tumours was noted (OR 1.18; 95% CI 1.12–1.24). Adjustment for serum levels of glucose and triglycerides did not alter these findings (results not shown). Association between GGT and ER− cancer was no longer seen, whilst others remained, in a sensitivity analysis only including cases with complete receptor information (results not shown).

### Case–case analysis

Cases were less likely to be PR− compared to PR+ with increasing log-transformed GGT (OR 0.87, 95% CI 0.80–0.95). A similar trend was seen for quartiles of GGT. No association was found for ER− cancers, with ER+ cancers as the referent.

**Fig. 1:**
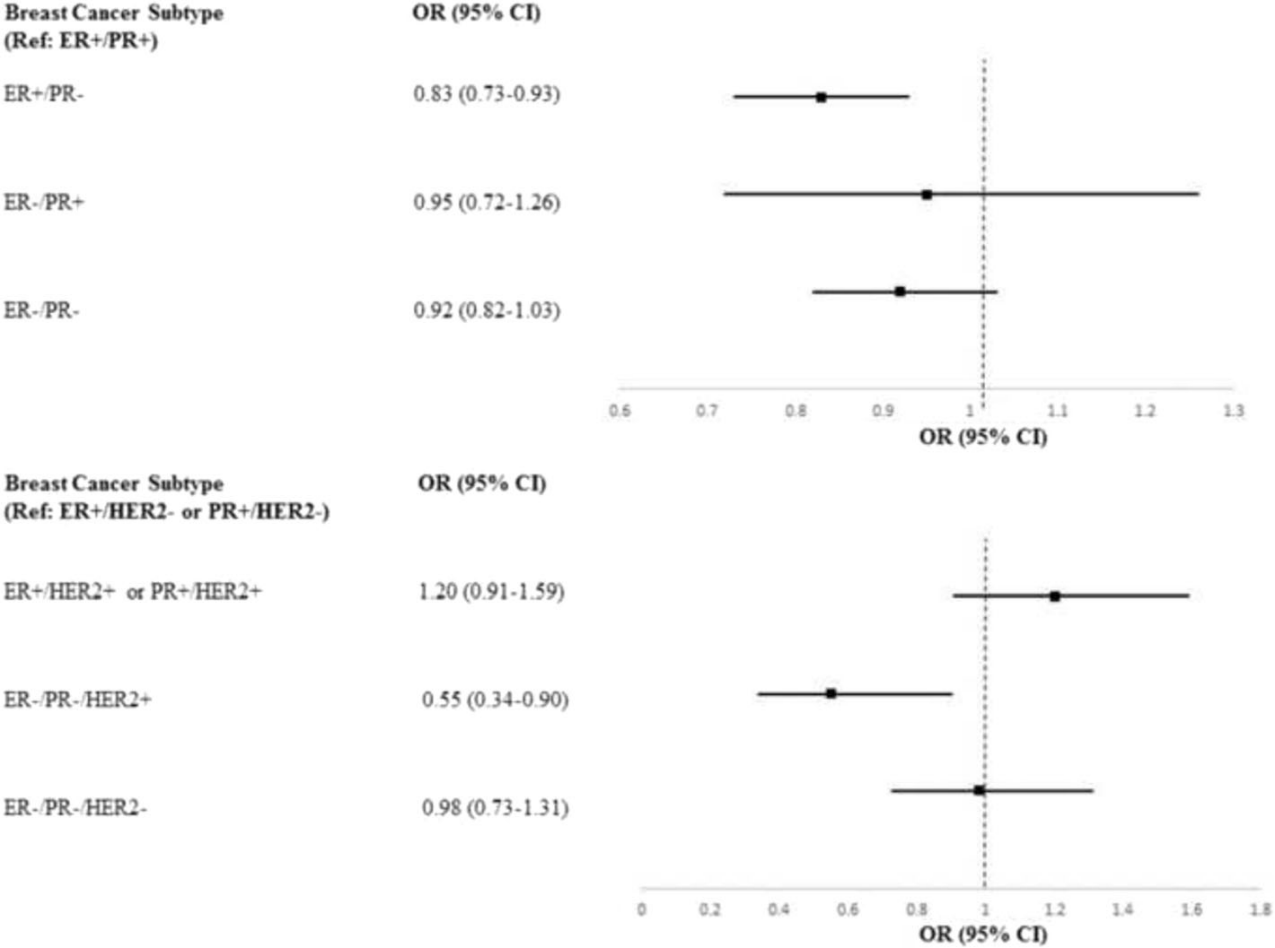
Multinomial logistic regression analysis for log-transformed levels of GGT with breast cancer subtype as outcome variable. ER+/PR+ and ER+/HER2− or PR+/HER2− assigned as reference values. All models were adjusted for age at diagnosis, menopausal status, socioeconomic status, education, parity, CCI, and interval between measurement and cancer diagnosis

We subsequently investigated any association between serum GGT and breast cancer subtypes based on the combination of ER/PR and ER/PR/HER2 status (Fig. 1). Compared to ER+/PR+ tumours, increasing GGT was associated with a lower odds of ER+/PR− tumours (OR 0.83, 95% CI 0.73–0.93 for each log unit increase in GGT). A similar but weaker trend was seen for ER−/PR− tumours (Additional file 1: Table S2). Associations were slightly weaker when limited to cases with complete information on the three receptors, e.g. OR for ER+/PR− tumours: 0.83 (95% CI 0.67–1.02) for each log-unit increase in GGT.

We also found an inverse trend between GGT and odds of being diagnosed with ER−/PR−/HER2+ cancers (OR 0.55, 95% CI 0.34–0.90 when compared to the referent group ER+/HER2− or PR+/HER2− breast cancers). No marked difference across GGT levels was observed for other subtypes. Additional adjustments for serum levels of glucose and triglycerides did not alter these observations (results not shown).

## Discussion

Increasing serum levels of GGT corresponded to increased odds of ER+, ER− and PR+ tumours but not related to higher risk of PR− breast cancers. Our findings when comparing different subtypes also suggested associations between GGT levels and specific breast cancer subtypes.

Oxidative stress may contribute to development of ER+ breast cancers by modifying the structure and function of redox-sensitive ERs on the cell surface, which reduces expression of an oxidant-sensitive set of oestrogen-inducible genes, including genes involved in cell growth, invasion, and PR expression [24]. This may correlate with suppression of the PR gene [24, 25]. We found that higher levels of GGT were associated with relatively attenuated odds of ER− subtype. These conflicting results might imply more complex underlying mechanisms. The negative association found between GGT and ER−/PR−/HER2+ breast cancers may support previous notions that HER2-overexpressing tumours have lower glutathione levels and GGT activity [26, 27]. However, this would require further confirmation in larger studies. It is also possible that this finding on HER2 was driven by the inverse association between GGT and PR− compared to PR+ breast cancers.

Our results may support distinct aetiological pathways preceding breast cancer subtypes, in particular PR− cancers. Previously, different associations with breast cancer subtypes have been reported with parity, first-time births, breastfeeding and oral contraceptive [11, 28, 29]. Obesity [11, 21–23] and dietary fat intake [30–32] may also affect subtype development via hormonal modulation, increased oxidative stress and inflammation [22]. Similar roles have been indicated for circulating glucose [17, 33, 34]. Therefore, increased GGT associated with increased risk of breast cancer may partly be acting as a marker of these metabolic disorders. Nonetheless, our results were unaltered when adjusted for serum glucose and triglycerides.

The strength of this study is the large number of women included with complete follow-up information. The AMORIS population is similar to the general working population of Stockholm in terms of socioeconomic status and ethnicity [5]. There was limited information on receptor status in earlier diagnoses. However, results were similar when limited to data with complete receptor information, i.e. diagnoses from 2007 onwards. Information on other risk factors such as hormone-replacement therapy, body mass index and alcohol intake was not available, which necessitates future studies incorporating this information. With respect to alcohol, however, the blood samples were collected prior to the major increase in alcohol use in women.

## Conclusion

Pre-diagnostic serum GGT levels are associated with specific female breast cancer subtypes. Given prior evidence showing increased GGT to be associated with other lifestyle-related disorders, it is important to consider GGT as a proxy of these factors. Understanding of this complex association may lead to mechanistic studies to confirm the role of oxidative stress in specific breast cancer subtypes, which may have clinical implications.

## Additional file

Additional file 1: Supplementary tables. (DOCX 39 kb)

## Abbreviations

CCI: Charlson co-morbidity index
ER: Oestrogen receptor
GGT: Gamma-glutamyl transferase
HER2: Human epidermal growth factor 2
OR: Odds ratio
PR: Progesterone receptor
