# Supplementary material for: Circulating gamma-glutamyl transferase and development of specific breast cancer subtypes: findings from the Apolipoprotein Mortality Risk (AMORIS) cohort
Source: Breast Cancer Res. 2017 Mar 6;19:22. doi: 10.1186/s13058-017-0816-7 (PMC5339947; doi:10.1186/s13058-017-0816-7)
Supplement: Additional file 1: — Supplementary tables. (DOCX 39 kb) [file 13058_2017_816_MOESM1_ESM.docx]

**Table S1**. Descriptive characteristics of study population

|  | **Cases** | **Controls** |
| --- | --- | --- |
| **Age (years)^*^** |  |  |
| Mean (SD) | 61.68(11.81) | 59.79(15.64) |
| **Socioeconomic Status, N(%)** |  |  |
| White Collar (high) | 4602(42.37) | 38392(35.35) |
| Blue Collar (low) | 5392(49.65) | 56337(51.87) |
| Unemployed/missing | 867(7.98) | 13881(12.78) |
| **Education Level, N(%)** |  |  |
| Lower than high school | 2961(27.26) | 29868(27.50) |
| High school | 4482(41.27) | 44932(41.37) |
| Higher education | 3105(28.59) | 26839(24.71) |
| Missing | 313(2.88) | 6971(6.42) |
| **Gamma Glutamyl Transferase (U/L)** |  |  |
| Quartile 1 N(%) | 2407(22.16) | 25674(23.64) |
| Quartile 2 N(%) | 2605(23.98) | 26457(24.36) |
| Quartile 3 N(%) | 2918(26.87) | 29059(26.76) |
| Quartile 4 N(%) | 2931(26.99) | 27420(25.25) |
| **Menopausal status at diagnosis, N(%)** |  |  |
| Premenopause | 1833(16.88) |  |
| Postmenopause | 6976(64.23) |  |
| Unknown | 2052(18.89) |  |
| **ER Status, N(%)** |  |  |
| Positive | 5939(54.68) |  |
| Negative | 1295(11.92) |  |
| Unknown | 3627(33.39) |  |
| **PR Status, N(%)** |  |  |
| Positive | 4938(45.47) |  |
| Negative | 2207(20.32) |  |
| Unknown | 3716(34.21) |  |
| **HER2 Status, N(%)** |  |  |
| Positive | 246(2.26) |  |
| Negative | 1951(17.96) |  |
| Unknown | 8664(79.77) |  |

^*^Age at diagnosis of cases and age at selection of controls

**Table S2.** Multinomial logistic regression analysis with breast cancer subtype as outcome variable. ER+/PR+ and ER+/HER2- or PR+/HER2- assigned as reference values. All models were adjusted for age at diagnosis, menopausal status, socioeconomic status, education, parity, CCI, and interval between measurement and cancer diagnosis.

|  | **Breast Cancer Subtype** | | | | | | |
| --- | --- | --- | --- | --- | --- | --- | --- |
| **GGT U/L** | **ER+/PR+ (Reference)** | **ER+/PR-** | | **ER-/PR+** | | **ER-/PR-** | |
|  | N | N | **OR (95% CI)** | N | **OR (95% CI)** | N | **OR (95% CI)** |
| GGT log |  |  | 0.83 (0.73-0.93) |  | 0.95 (0.72-1.26) |  | 0.92(0.82-1.03) |
| 0-11.40 | 1037 | 253 | 1 (ref) | 30 | 1 (ref) | 258 | 1 (ref) |
| 11.40-15.00 | 1129 | 274 | 0.94(0.78-1.14) | 46 | 1.45(0.91-2.33) | 256 | 0.89(0.74-1.01) |
| 15.00-21.60 | 1280 | 286 | 0.85(0.70-1.03) | 44 | 1.23(0.77-1.99) | 293 | 0.90(0.74-1.09) |
| ≥21.60 | 1329 | 279 | 0.73(0.60-0.89) | 42 | 1.19(0.73-1.95) | 304 | 0.87(0.72-1.05) |
| P_trend_ |  |  | 0.0008 |  | 0.74 |  | 0.19 |
| **GGT U/L** | **ER+/HER2- or PR+/HER2- (Reference)** | **ER+/HER2+ or PR+/HER2+** | | **ER-/PR-/HER2+** | | **ER-/PR-/HER2-** | |
|  | N | N | **OR (95% CI)** | N | **OR (95% CI)** | N | **OR (95% CI)** |
| GGT log |  |  | 1.20(0.91-1.59) |  | 0.55(0.34-0.90) |  | 0.98(0.73-1.31) |
| 0-11.40 | 415 | 36 | 1 (ref) | 37 | 1 (ref) | 39 | 1 (ref) |
| 11.40-15.00 | 458 | 39 | 1.01(0.63-1.63) | 14 | 0.34(0.18-0.64) | 47 | 1.08(0.69-1.69) |
| 15.00-21.60 | 489 | 41 | 1.04(0.65-1.67) | 13 | 0.30(0.16-0.57) | 43 | 0.92(0.58-1.46) |
| ≥21.60 | 414 | 46 | 1.48(0.92-2.38) | 16 | 0.45(0.24-0.83) | 40 | 1.02(0.64-1.64) |
| P_trend_ |  |  | 0.11 |  | 0.002 |  | 0.88 |

**Figure S1**. Frequency of each breast cancer tumour subtype in the study population

All tumours

N= 10,861

ER+ or PR+

N= 6,029

ER-/PR-

N=1,111

HER2-

N= 1,776

HER2+

N= 80

HER2+

N= 162

HER2-

N= 169

**Subgroup**

**ER+/HER2- or PR+/HER2-**

**ER+/HER2+or PR+/HER2+**

**ER-/PR-/HER2+**

**ER-/PR-/HER2-**
